# Heterogenous Immune Response to TB is Not a False Negative

**DOI:** 10.4269/ajtmh.19-0896b

**Published:** 2020-03

**Authors:** Mao-Shui Wang, Xin-Jie Liu

**Affiliations:** Department of Pediatrics; Qilu Hospital; Shandong University; Jinan, China; Department of Lab Medicine; Shandong Provincial Chest Hospital; Jinan, China; E-mail: wangmaoshui@gmail.com; Department of Pediatrics; Qilu Hospital; Shandong University; Jinan, China; E-mail: liuxinjie@sdu.edu.cn

Dear Sir,

Thank you for your interest. A false negative is a test result that indicates that a condition does not hold, although in fact it does.^1^ Because the interferon-gamma release assay (IGRA) is designed for the diagnosis of tuberculosis (TB) infection, the confirmed TB patients with IGRA (−) should be considered to have a false-negative result. However, your comments are very interesting for us, and we have some data that may help answer your questions.

First, a poor agreement between repeated T-SPOT.TB in a short time period was observed. Reversions and conversions were reported in about 15% of TB patients undergoing serial testing.^2^ In a recent review, Banaei et al.^3^ summarized analytical and immunological factors associated with false-negative results. These suggested that false-negative IGRA results are common in clinical practice and that analytical errors cannot be eliminated.

Second, between November 2018 and September 2019, invalid results were reviewed in our laboratory and seen in 4.4% (66/1,503) of tests. These invalid results were more likely due to a weak mitogen (positive, *n* = 60) control response than a robust nil (negative, *n* = 6) control response. The data are inconsistent with those reported by Mandalakas et al.^4^ In our opinion, this paradox cannot be fully explained by analytical errors, and genetic or immunological factors may be responsible for this difference.

Third, in a recent study, Verrall et al.^5^ found that early clearance of *M. tuberculosis* is associated with increased innate immune responses. Likewise, the clearance of *M. tuberculosis* may be associated with T-cell responses. We evaluated the association between relapse and IGRA results and found that patients with a negative IGRA result have a high risk of relapse. However, we think the data are not solid because of a high proportion of lymph node TB reported in China.^6^ In fact, most lymph node TB in children is caused by BCG vaccination, and this may have biased our results.

Fourth, in two meta-analyses,^7,8^ it was demonstrated that 1) reversion from positive to negative IGRA values occurs in a minority of treated patients; 2) variations in IGRA results between participants are large, and hence, IGRAs are unlikely to be useful for monitoring anti-TB therapy. Therefore, treatment may have a limited role in influencing the IGRA result. In our study, the period between T-SPOT.TB test and initiation of anti-TB therapy was not considered as a criterion for enrolling patients. However, 147 (94.2%) patients were tested for T-SPOT.TB before the initiation of anti-TB therapy, seven (4.5%) were tested within 7 days of initiating therapy, and only two were tested > 7 days after therapy initiation. Therefore, in our study, anti-TB therapy should not be considered a risk factor for false-negative IGRA results.
